# A Rare Case of Atraumatic Splenic Rupture Due to Chronic Pancreatitis

**DOI:** 10.7759/cureus.19936

**Published:** 2021-11-27

**Authors:** Rita Martelo, João C Morais, Angeles Rábago, Inês C Borges, Francisco Rodrigues

**Affiliations:** 1 General Surgery, Hospital Vila Franca de Xira, Vila Franca de Xira, PRT

**Keywords:** splenic vein thrombosis, laparotomy, splenectomy, pancreatitis, splenic hematoma, atraumatic splenic rupture, spleen

## Abstract

Atraumatic splenic rupture is a rare but dangerous complication of chronic pancreatitis, vastly ignored in emergency literature. The anatomical relationship between the spleen and the tail of the pancreas contributes to the pathophysiology when an inflammatory process is in progress, although the mechanisms are not fully understood. The authors report the case of a 41-year-old male, previously undiagnosed with chronic pancreatitis, presenting with atraumatic splenic rupture. Due to worsening abdominal pain and hemodynamic instability, he underwent total splenectomy. The final diagnosis was obtained through contrast-enhanced abdominal computed tomography scans, intraoperative findings and histopathological examination of the surgical specimen, as frequently reported in previous cases. Total splenectomy is the treatment of choice, as the failure rate of the conservative approach is high. Few of these cases are described and a deeper understanding of the subject is needed. As this condition can worsen in a short time, a prompt diagnosis followed by adequate treatment can impact the morbidity and mortality associated with splenic rupture. High clinical suspicion is essential and increased knowledge about the pathophysiology and presentation of splenic complications in pancreatitis may alert emergency physicians to these fatal complications.

## Introduction

Atraumatic splenic rupture (ASR) is rare, although being a well-documented injury in the setting of blunt abdominal trauma. This diagnosis is often overlooked in a patient without a history of trauma but it is of utmost importance to be done in due time since it is a life-threatening condition [1]. Acute or chronic pancreatitis is a documented cause of ASR, although its incidence is unclear. It occurs because of the anatomical proximity between the pancreatic tail and the spleen via direct erosion of pseudocysts, localized portal hypertension secondary to splenic vein thrombosis, acute inflammation of intrasplenic ectopic pancreatic tissue, tryptic erosion in the splenic hilum or perisplenic adhesions that fix the spleen due to recurrent pancreatitis and perisplenitis [2-4].

We present the case of an ASR in a male patient not previously diagnosed with chronic pancreatitis. Due to its rarity, the understanding of these particular cases is sparse, making it fundamental for emergency surgeons to be aware of this entity.

This article is a development of a previously presented poster at the XLI Congresso Nacional de Cirurgia on June 18, 2021.

## Case presentation

A 41-year-old man, with a history of alcohol-induced acute pancreatitis eight months before, presented to the emergency department of Hospital Vila Franca de Xira. He complained of nausea, vomiting and severe abdominal pain in the left upper quadrant radiating to his left shoulder for two days. He also referred to anorexia, unquantified weight loss and night sweats in the three previous weeks. There was not any history of significant abdominal trauma.

In his previous hospitalization, he had elevated serum amylase and lipase levels. Imaging revealed a normal homogeneous liver with the absence of gallstones, a normally sized pancreas with regular contours and a normal-sized spleen with homogeneous echotexture. He was treated conservatively, abandoned alcohol consumption and recovered uneventfully, maintaining sporadic abdominal pain in the left upper quadrant since then.

On this admission, the patient was hemodynamically stable, afebrile, oxygen saturation at 97% on room air and the abdominal examination revealed moderate upper abdominal pain but no rebound tenderness. The laboratory test results were remarkable for white blood cell count (19.5×103/μL) and CRP (4.95 mg/dL). The serum hemoglobin level was normal (14.5 g/dL), as well as the platelet count, prothrombin time and serum bilirubin, amylase, lipase, lactate dehydrogenase and haptoglobin levels. There was no evidence of red blood cell destruction.

**Figure 1:**
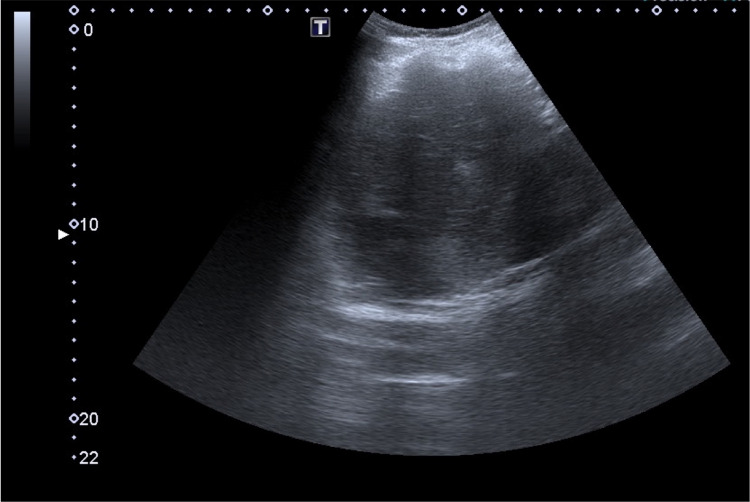
Abdominal ultrasound scan on admission showing spleen with heterogeneous echotexture and poorly defined contours.

**Figure 2:**
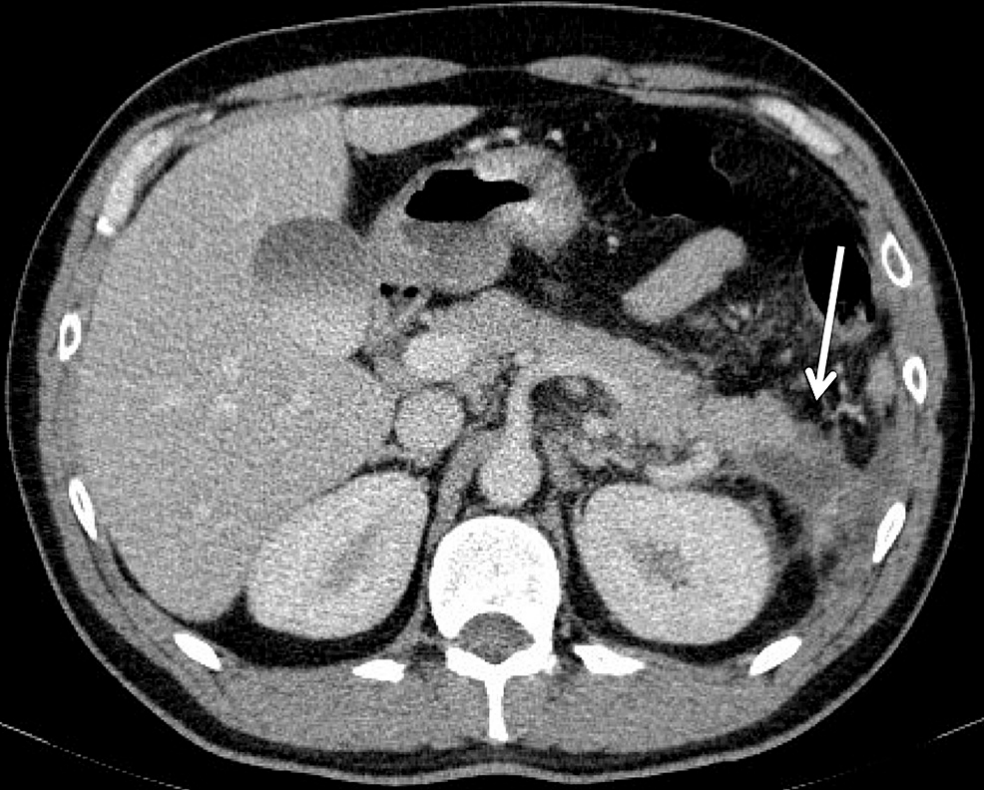
Contrast-enhanced CT scan (axial plane) on admission showing irregular margins on the tail of the pancreas in contact with a collection that extend into the anterior pararenal space.

**Figure 3:**
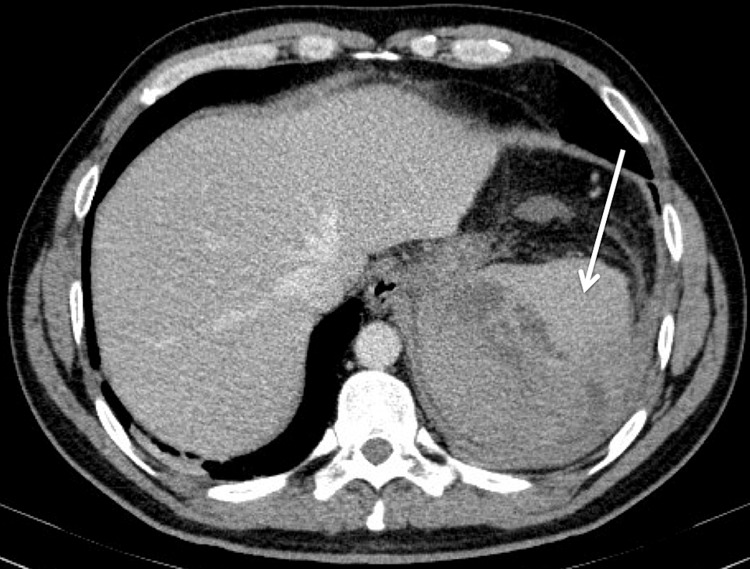
Contrast-enhanced CT scan (axial plane) on admission showing heterogeneous splenomegaly with several hyperdense areas, corresponding to intrasplenic and subcapsular hematomas together with rupture of the splenic capsule.

An abdominal ultrasound scan revealed a heterogeneous spleen with imprecise borders, with a liquid collection of undetermined etiology in subphrenic topography. The liver had normal dimensions, regular contours and homogeneous echotexture (Figure 1). The ultrasound scan was complemented with a contrast-enhanced abdominal computed tomography (CECT) scan with confirmation of large heterogeneous splenomegaly (13.5 cm), with several areas of greater density, densification of adjacent fat and a liquid collection, measuring 34 x 22 mm, between the tail of the pancreas and the splenic hilum, suggestive of spontaneous intrasplenic rupture. The pancreas was globally homogeneous except in the tail, where it presented irregular margins in contact with the liquid collection (Figures 2, 3).

**Figure 4:**
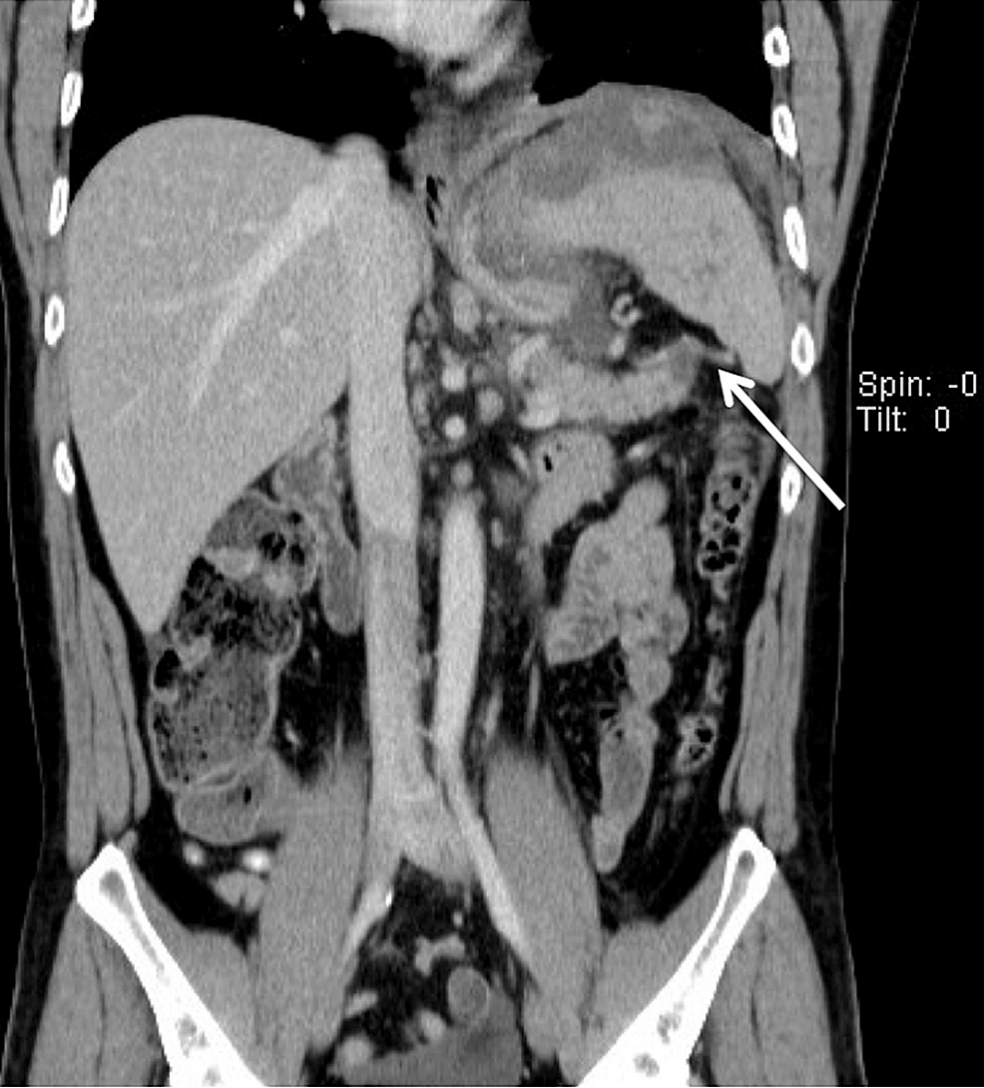
Contrast-enhanced CT scan (coronal plane) 24 hours after admission showing the heterogeneous collection that extends along the subphrenic space, perisplenic region and pancreatic tail.

**Figure 5:**
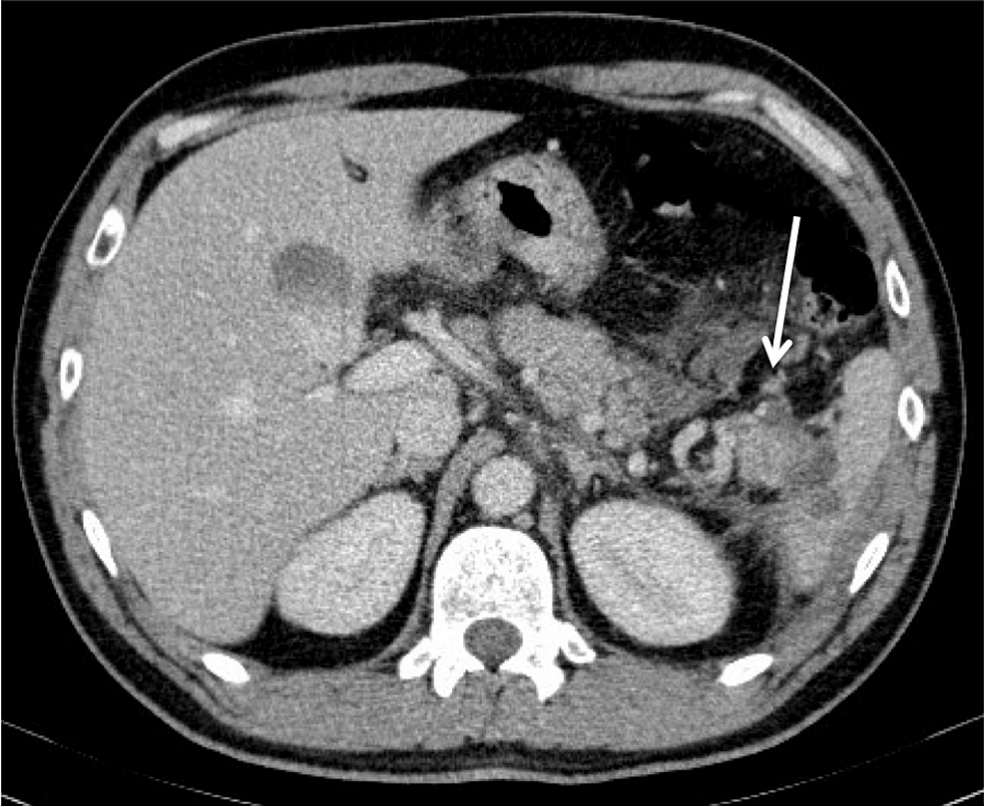
Contrast-enhanced CT scan (axial plane) 24 hours after admission showing splenic vein thrombosis.

Over the following 24 hours, although being treated with fluid resuscitation and antibiotic administration, the patient became tachycardic, with fever and signs of peritonism. The hemoglobin level dropped to 12.8 g/dL and the inflammatory parameters worsened. Since his serial serum amylase levels remained within the normal range, the diagnosis of acute pancreatitis became less likely. A new CECT scan was performed, which revealed worsening of the free heterogeneous fluid, not clearly hematic, in perigastric and perisplenic location, involving the tail of the pancreas and in the pelvic cavity (Figure 4). The heterogeneous splenomegaly persisted and thrombosis of the splenic vein was identified. The pancreas remained normal in size and morphology, with no dilatation of the duct of Wirsung (Figure 5).

The patient was transfused with a unit of packed red blood cells and proposed an exploratory laparotomy to which he consented. Intra-operatively we found an infected hemoperitoneum, splenomegaly with multiple subcapsular hematomas and disintegration of the parenchyma in the inferior pole, due to the rupture of one of the subcapsular hematomas. A total splenectomy was performed.

Until the pathology report came, we also excluded infectious mononucleosis, infective endocarditis, sarcoidosis, symptomatic human immunodeficiency virus infection, systemic lupus erythematosus or myeloproliferative disorders. The patient presented a normal erythrocyte sedimentation rate, a normal peripheral blood smear and a normal bone marrow biopsy.

**Figure 6:**
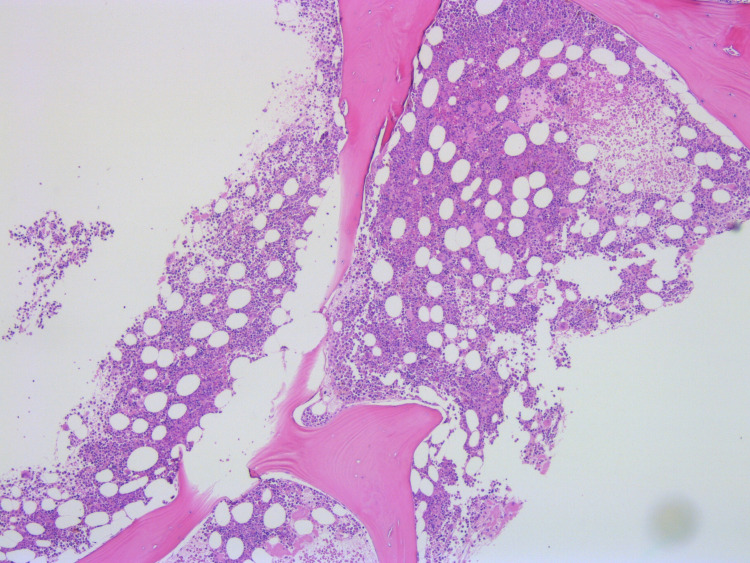
Histological features of the spleen specimen (hematoxylin-eosin staining).

Histopathological examination of the spleen specimen was compatible with the first diagnosis and identified fragments of pancreatic parenchyma with fibrosis, acinar atrophy and irregular ducts (Figure 6). These pancreas fragments infiltrated the splenic parenchyma at the site of capsular rupture, where hemorrhage and coagulation necrosis occurred. This established the diagnosis of chronic pancreatitis as the cause of the ASR.

On the seventh day of post-op, the patient developed anorexia and pain in the right lower quadrant and an abdominal computed tomography (CT) scan revealed an acute appendicitis. He was submitted to a relaparotomy and an appendectomy was performed. The patient fully recovered from the second surgery and was discharged, free of complications, five days later.

## Discussion

The spleen remains the most frequently affected organ in blunt trauma, accounting for up to 50% of all abdominal solid organ injuries [5]. There are several documented cases of traumatic splenic rupture (TSR) in the scientific literature [6] as well as guidelines on its treatment [7]. Meanwhile, a splenic rupture in the absence of trauma or a diseased spleen is a rare phenomenon, although being a life-threatening one [8,9]. The incidence of ASR has not yet been clarified and related guidelines or a standard of care regarding the diagnosis and treatment is still lacking [9]. These cases often go unnoticed, because of the absence of historical clues at presentation, highlighting the importance for emergency physicians to bear this option in mind [8]. Renzulli et al. found that in 51.2% of the cases the underlying cause for the ASR was not suspected until after hospital presentation [8]. In our clinical case, imaging and laboratory tests did not support the clinical hypothesis of chronic pancreatitis and though the splenic rupture was readily recognized, we could not identify its cause until the histopathological examination of the surgical specimen. In their systematic review, Renzulli, et al. also pointed out the six main etiological factors for ASR as idiopathic (6.4%) and neoplastic (30.3%), infectious (27.3%), inflammatory (20.0%), drug/treatment-related (9.2%) and mechanical (6.8%) disorders [8]. Until 2009, only 65 cases of splenic rupture due to chronic pancreatitis were reported in the literature, comprising 8.27% of all occult splenic ruptures [6].

The pathophysiological mechanisms of splenic rupture in chronic pancreatitis are not fully understood [3]. The distal portion of the pancreatic tail reaches the splenic hilum, along with the splenic vessels, within the two layers of the peritoneum that form the splenorenal ligament [3,10]. It has been hypothesized that when a leak occurs, enzyme-rich pancreatic fluid gains straight access to the splenic hilum and capsule, injuring the splenic vessels and splenic parenchyma. This can lead to various complications such as splenic vein thrombosis, intrasplenic pseudocysts, splenic rupture, infarction, necrosis, splenic hematoma and bleeding from eroded splenic vessels [3,11]. The described complications are present in 1%-5% of the cases of pancreatitis. Splenic rupture is the second most prevalent, accounting for 36% of all complications, after splenic vein thrombosis [11-13]. The majority of patients with complications are men, median age 39 and alcohol is the main etiology (67.9% to 84.6% of the cases), as in our case report. Splenic rupture is also more common in chronic than in acute pancreatitis, within the first two years of pancreatic disease and is often associated with occlusion of the splenic vein, a pseudocyst or necrosis in the tail of the pancreas [2,10,12]. Splenic vein thrombosis may have been the factor that precipitated ASR in our clinical case. The patient had no intrasplenic ectopic pancreatic tissue on histological examination, nor perisplenic adhesions or a pancreatic pseudocyst on CT scan or laparotomy. The precise mechanism of thrombosis is probably multifactorial, including prothrombotic, local inflammatory transformations in the endothelium, low pancreatic perfusion and extrinsic damage secondary to venous compression from adjacent pseudocysts, edema, enlarged retroperitoneal lymph nodes or even fibrosis that appear later in the course of the pancreatic disease [3,11,14]. Splenic vein thrombosis, when associated with inflammation in the tail of the pancreas, appears to greatly increase the likelihood of splenic complications [10].

Clinical findings of ASR are nonspecific, challenging the diagnosis that relies mostly on imaging studies. The commonest symptom is abdominal pain in the left upper quadrant, radiating to the left shoulder (Kehr’s sign) in 50% of the cases, followed by nausea or vomiting and pain in the left chest due to pleural effusion. A decrease in serum hemoglobin level can also be verified as well as normal serum amylase, lipase and troponin levels [2,9,11,15,16]. The scarcity of symptomatology emphasizes the importance of CT scans for the diagnosis. This should be the method of choice when clinical suspicion is present [10]. The reported sensitivity of ultrasound and CECT in diagnosing ASR is 57.1% and 85.7%, respectively [9]. Although magnetic resonance imaging is better at characterizing soft tissues and vascular changes, a CT scan is faster in assessing the degree of hemorrhage, vascular involvement and fluid collections [2,9,15]. ASR should be suspected if a patient with abdominal pain is hemodynamically unstable and the spleen is swollen on a CT scan. In a series of eight ARS cases, all spleens were swollen and hematomas were found in all but one patient, suggesting that the spleens were previously diseased and this presentation may result from the development and rupture of a hematoma [9].

Splenic vein thrombosis and probable segmental portal hypertension present an increased risk of splenic rupture and nonsurgical management can be deleterious [3,12]. The treatment of ASR should be guided according to hemodynamics, the amount of blood product used, advanced age, anticoagulant treatment, etiological factors and the degree of hemoperitoneum or extension of splenic injury in CT scans [2,8].

Whenever the hemodynamic status is stable and the etiological factors are evident, the conservative approach, with percutaneous drainage and/or splenic artery embolization, can be weighted, provided that there is strict monitoring with serial ultrasound or CT scans [2,13,15,17,18]. Arterial embolization creates an area of infarction of less than 50% of the splenic volume and can be applied for both etiological diagnosis and treatment of bleeding. However, its role is limited due to its association with a splenic abscess in 25% of cases, with only a few published cases and no long-term results [16]. Even if all preconditions for non-operative management are met, the success rate is low, requiring frequently emergent surgery. The overall splenic salvage rate is 13.7% [8,9].

Hemodynamically unstable patients will need an emergency laparotomy to perform a total splenectomy or distal pancreatosplenectomy, which may lessen the risk of pancreatic fistula or leak [3,10,12]. This approach offers the advantage of a pathological examination of the spleen and other abnormalities that can help uncover the underlying disease, as in our case report [14]. Furthermore, a considerable number of malignancies can cause ASR, prohibiting any organ-preserving approach. Finally, in most patients with ASR, the immune function of the spleen may have already been compromised by the alteration or infiltration of the splenic parenchyma, resulting in functional hyposplenism, thereby decreasing the risk of a post-splenectomy infection [2,8,9].

In this case report, the patient's clinical worsening led to the need for urgent surgery. A laparotomy was performed due to hemodynamic instability and the possibility of finding severe adhesions that could complicate a laparoscopic procedure. According to the literature, more than 90% of the ruptured cases were managed with laparotomy and splenectomy [2,3]. Renzulli, et al. reported that the main approach was total splenectomy in 84.4% of the cases, followed by conservative treatment (with or without transcatheter arterial embolization) in 14.7% and organ-preserving surgery in 1.2%. The last two options were chosen especially in patients with ASR of non-malignant etiology and 17% ultimately had a late splenectomy because of re-bleeding. The number of patients undergoing organ-preserving surgery or nonsurgical management is low compared to those with blunt abdominal trauma, thus suggesting that treatment guidelines for TSR cannot simply be applied to ASR [8].

ASR has greater surgical difficulty and a poorer outcome than TSR [9]. Reported mortality of ASR ranges from 12.2% to 20%, being splenomegaly, age over 40 years, neoplastic diseases and late diagnosis or treatment, significant negative prognostic factors [8,9,13,17]. Especially in pancreatitis, splenic complications are associated with higher morbidity (79%) and important mortality (8%), when compared to morbidity and mortality rates of 39% and 3.5% in patients deprived of splenic involvement [10]. Early detection and intervention are essential for optimal patient outcomes [2].

## Conclusions

ASR is a rare and dangerous complication of chronic pancreatitis that can potentially lead to a lethal outcome. Splenic parenchyma complications are increasingly being documented in pancreatitis and should be considered in patients with inflammation or necrosis of the tail of the pancreas. Diagnosis is challenging and requires a high index of suspicion. Contrast-enhanced computed tomography scan is the method of choice and intraoperative examination is helpful in discovering underlying abnormalities. Exploratory laparotomy with splenectomy remains the first-line treatment in most cases.

Knowledge about this condition is insufficient. Clinical reports such as the one presented are important to remind emergency physicians that a ruptured spleen can occur in the absence of major trauma or previous splenic disease. Prompt recognition and intervention are essential to change the associated high morbidity and mortality.
